# Periodontal status associated with dual habits of smoking and smokeless tobacco use: A cross-sectional study in young adults

**DOI:** 10.34172/japid.2021.010

**Published:** 2021-07-13

**Authors:** Abdul Ahad, Afshan Bey, Saif Khan, Mohammad Sami Ahmad

**Affiliations:** ^1^Department of Dentistry, Medini Rai Medical College, Palamu, Jharkhand, India; ^2^Department of Periodontics, Dr. Ziauddin Ahmad Dental College, Faculty of Medicine, Aligarh Muslim University, Aligarh, India; ^3^Department of Preventive Dental Sciences, College of Dentistry, Taibah University, Madinah, Saudi Arabia

**Keywords:** Gingival recession, periodontitis, smokeless tobacco, smoking

## Abstract

**Background:**

Tobacco smoke is an established risk factor for periodontitis. However, few studies have evaluated the periodontal status of smokeless tobacco (SLT) users, while that of individuals with dual habits has largely been unexplored. Therefore, the current study aimed to find if the periodontal status in individuals with dual habits of smoking and SLT use is different from those with any single habit.

**Methods:**

Four groups (A: exclusive smokers, B: exclusive tobacco chewers, C: individuals with dual habits, and D: non-users of tobacco), each comprising 75 males in the age group of 20 to 35 years, were selected. Along with the history of tobacco use, a modified oral hygiene index (OHI), gingival index (GI), probing depth (PD), and the number of teeth with gingival recession (GR) were recorded. The data were assessed using the Chi-squared test, one-way ANOVA, and logistic regression. Statistical significance was set at P<0.05.

**Results:**

Group C exhibited the highest mean OHI scores, with 94.66% of participants having poor oral hygiene (OHI>3.0). The prevalence of severe gingivitis (GI>2.0) was significantly lower among exclusive smokers (group A) and those with dual habits (group C) compared to the other two groups. As much as 60% of group C participants had average PD in the range of 4-6 mm, while deeper average PD (>6 mm) was most common among smokers. The highest risk of having a tooth with GR was also associated with the dual habit (OR = 4.33, 95% CI = 3.24 - 5.76) compared with the non-users.

**Conclusion:**

While both forms of tobacco were associated with poor periodontal status, the additive effect of smoking and SLT use was evident in almost all the parameters, more so with poor oral hygiene and the prevalence of gingival recession. These findings emphasize that individuals with dual habits have an additional risk for periodontal destruction.

## Introduction

The direct or indirect association of tobacco use with various diseases has been established nowadays, and the list of these diseases is continually growing. Tobacco consumption is linked with malignancy of the oropharyngeal region.^1^ It has also been linked to cardiovascular diseases, preterm and low birth weight, and periodontal disease.^2-4^ It is now one of the major avoidable causes of morbidity and mortality worldwide.^5,6^ The use of tobacco has declined in developed countries due to solid control measures. For example, in the USA, the prevalence of smokeless tobacco (SLT) use has been reported to be as low as 5.6%.^7^ However, in third world countries, its use has significantly increased over the past few decades, particularly among young adults.^8,9^ Currently, South Asia faces the biggest brunt of health issues attributable to SLT products, resulting in a significant financial burden on tobacco users, their families, and governments.^10^ Tobacco is used for smoking and chewing in various forms. Both these habits are very common in the Indian subcontinent.^8^ Approximately 17% of the total population in Southeast Asia is reported to use oral tobacco, of which 82% belong to India, where SLT accounts for 35–40% of total tobacco consumption.^11^

Periodontitis results from a complex interrelationship between the host and microbes. Risk factors such as smoking can modify the expression and progression of this disease. While the effect of smoking on periodontal disease has been investigated in many studies and a positive correlation has been found, reports on the effect of SLT use on the periodontium are comparatively few, with varying results.^12,13^

The South Asian population consumes tobacco in a variety of forms. In north India, the most commonly used products are beedis (tobacco wrapped in the dried leaves of *Bauhinia racemosa*) and cigarettes for smoking. In contrast, gutkha (a mixture of tobacco, betel nut, and some spices) and khaini (tobacco with lime) are the most common among the smokeless forms.^4,8,14^ Most of their users are males from lower socioeconomic backgrounds and lack awareness regarding oral health.^4,14^ Recent data shows that almost half of the adults who smoke beedis also use other forms of tobacco, including SLT products.^14^ This large proportion of the population who uses tobacco for both smoking and chewing has rarely been evaluated for the effect of a combination of these habits on the periodontium.^13^ It is important to find whether these individuals are at higher risk than those with a single habit of either smoking or tobacco chewing to deteriorate their periodontal health.

The association of smoking with the prevalence and severity of periodontal diseases has been well documented. Smokers are reported to have high levels of plaque and calculus compared to non-smokers.^15^ When the gingival response to plaque in the form of bleeding is considered, most studies have reported that smokers present with fewer bleeding sites on probing than non-smokers.^16,17^ As far as periodontitis is concerned, there is convincing evidence that periodontal pockets, clinical attachment loss, and alveolar bone loss are more prevalent and severe in smokers than non-smokers.^18^

On the other hand, most studies found no clear association between smokeless tobacco use and periodontal disease except for the significant gingival recession and attachment loss.^19-22^ Fisher et al reported a positive correlation between smokeless tobacco use and severe periodontal disease when the results were adjusted for confounding factors, such as age, gender, smoking, glycemic status, and dental visits in the past year.^17^ They found that smokeless tobacco users who never smoked were twice as likely to have severe active periodontal disease than individuals not using smokeless tobacco. Some studies found no clear relation between smokeless tobacco use and a higher risk for periodontitis.^23^ Kulkarni et al presented one of the few studies comparing smokers’ periodontal status with smokeless tobacco users. However, they excluded all subjects with dual habits.^4^

The present study aimed to find if young adult males with dual habits are more likely to have poor periodontal status than individuals with only a single habit of either smoking or smokeless tobacco use.

## Methods

This cross-sectional study was conducted following the principles outlined in the Declaration of Helsinki for medical research involving human subjects, as revised in 2008. The study protocol was assessed and approved by the institutional ethics committee. The sample size estimation was carried out after conducting a pilot study on 20 subjects. Considering a precision value (d) of 5%, confidence interval of 95%, and 0.05 as the significance level, the minimum sample size was estimated at 288 using the following formula: n = 4Pq/d^2^ where n is the total sample size, P is the expected prevalence percentage derived from the pilot study and q = 100-P.

### Inclusion criteria

Smokers (Group A) comprised subjects who had been smoking beedi or cigarettes at least once daily in the last year. In contrast, tobacco chewers (Group B) included non-smoking subjects who had been taking tobacco in the form of khaini or gutkha at least once daily in the last year. Group C (dual habit) comprised the subjects who had been smoking beedi or cigarettes and chewing tobacco in the form of khaini or gutkha at least once daily in the last year. Group D (no habit) comprised subjects who reported to have never used tobacco in any form.

### Exclusion criteria

Females, past smokers or tobacco chewers, individuals with <20 natural teeth, those with any chronic systemic disease, and those who received any periodontal treatment or antibiotics in the past three months were excluded.^24^ While counting the number of teeth, partially erupted third molars, any prosthesis or teeth with full coverage restorations were not considered.

### Recruitment of participants

Male patients in the age group of 20 to 35 years, reporting to the Department of Periodontics during the study period (2010 to 2016), were screened as potential participants for any of the four groups. All the patients who fulfilled the inclusion and exclusion criteria of any group and agreed to sign the informed consent forms were included. Seventy-five participants were included in group D after screening only 122 patients. However, due to strict criteria for the remaining groups and the refusal of some patients to provide consent, we had to screen a total of 1336 patients to recruit 75 participants in each group.

### Data collection

Demographic details followed by medical history, dental history, and history of tobacco use were recorded in a multiple-choice questionnaire. Clinical examination was performed using a dental mirror, a sharp explorer, and a calibrated periodontal probe (UNC-15, Hu-Friedy, Chicago, IL, USA) by two examiners blinded to the participant’s history of tobacco use. Both examiners evaluated clinical parameters in 15 patients before conducting the study. They showed high inter-examiner reliability (Cohen’s kappa = 0.9). The participants’ oral hygiene status was evaluated using a modified version of the Oral Hygiene Index (OHI). In this modification, all the fully erupted natural teeth were examined instead of one tooth per segment, while the scoring criteria for debris and calculus remained the same as described by Greene and Vermillion.^25^ Periodontal disease status was recorded by evaluating six aspects (mesiobuccal, mid-buccal, distobuccal, mesio-oral, mid-oral, and disto-oral) of each natural tooth using the following measures: gingival index (GI),^26^ probing depth (PD) in millimeters (mm), and the presence of gingival recession (GR). Teeth were counted as having gingival recession if the gingival margin was found at least one mm away from the cementoenamel junction (CEJ) on any aspect.

### Data analysis

Data were collected separately for four groups and presented as the percentage, mean, and standard deviation. SPSS 17.0 was used for statistical analyses. For categorical data, the intergroup comparison was made using the Chi-squared test. One-way ANOVA with post hoc Tukey test was used for pair-wise comparisons between the groups. Logistic regression analysis was performed to calculate Odd’s ratio for each group of tobacco users (groups A, B, and C) in comparison to the group of individuals without any tobacco use (group D). Statistical significance was defined at P<0.05.

## Results

Seventy-five individuals in each group underwent history recording by answering multiple-choice questions followed by a comprehensive periodontal examination. A total of 45864 sites in 7644 teeth were evaluated in 300 individuals. The data obtained from history recording are presented in Table 1, where a statistically significant difference was observed between the four groups for all the parameters. Group C had a significantly lower number of teeth (23.85±3.37) than any other group. Oral hygiene practices of individuals with dual habits were poorer than any other group, as only 65.33% of them used a toothbrush, while others used different types of chewing sticks and miswak. They were also careless in terms of the frequency of cleaning teeth, as only 17.33% cleaned their teeth twice daily. More than 30% of tobacco users, irrespective of the type of tobacco consumed, had no previous dental visits, compared to only 16% in the control group.

**Table 1:** Demographic data of the participants

**Variables**	**A** **(Smokers)**	**B** **(Tobacco chewers)**	**C** **(Dual habit)**	**D** **(No habit)**	**P-value**
**Age** (Mean ± SD)
Age in years	28.15±3.80	27.48±4.44	28.81±4.63	26.35±4.28	0.004*
**Number of teeth** (Mean ± SD)
Number of teeth	25.36±2.64	25.62±2.65	23.85±3.37	27.08±2.99	<0.001*
**Method of cleaning teeth** n (%)
Using a toothbrush	68 (90.66)	58 (77.33)	49 (65.33)	70 (93.33)	<0.001*
Conventional methods	7 (9.33)	17 (22.66)	26 (34.66)	5 (6.66)	
**Frequency of cleaning teeth** n (%)
Twice daily	15 (20)	23 (30.66)	13 (17.33)	43 (57.33)	<0.001*
Once-daily	59 (78.66)	50 (66.66)	59 (78.66)	31 (41.33)	
Occasionally	1 (1.33)	2 (2.66)	3 (4)	1 (1.33)
**History of the past dental visit** n (%)
Within last one year	6 (8)	4 (5.33)	6 (8)	7 (9.33)	0.039*
More than one year back	41 (54.66)	40 (53.33)	46 (61.33)	56 (74.66)	
Never visited a dentist	28 (37.33)	31 (41.33)	23 (30.66)	12 (16)

*Statistically significant (P<0.05). P-values for age and the number of teeth were obtained using the one-way ANOVA and chi-squared test for all other parameters.

### Oral hygiene index

The data from the clinical examination are summarized in Table 2. The oral hygiene score of each individual was calculated as the sum of the debris score and calculus score. Oral hygiene status was further classified as good (0.0 to 1.2), fair (1.3 to 3.0), and poor (3.1 to 6.0) as presented and compared in Table 3. On inter-group comparison, the values of group C (dual habit) were found to be significantly higher (P<0.05) than all other groups (Table 2 and 4).

**Table 2:** Data from clinical evaluation of periodontal parameters

**Parameters** (Mean ± SD)	**A** **(Smokers)**	**B** **(Tobacco chewers)**	**C** **(Dual habit)**	**D** **(No habit)**	**P-value**
OHI	3.37±1.09	3.12±1.11	4.13±0.74	3.04±1.08	<0.001*
GI	1.09±0.49	1.42±0.55	1.32±0.67	1.61±0.52	<0.001*
PD	4.66±1.23	3.95±0.81	4.48±0.82	3.33±0.77	<0.001*
GR	1.91±0.74	2.8±1.03	3.08±1.24	1.31±0.96	<0.001*

*Highly significant (P<0.001). P-values were obtained using one-way ANOVA. OHI: Oral Hygiene Index, GI: Gingival Index, PD: Probing depth (in mm), GR: Number of teeth with gingival recession.

**Table 3:** Distribution of participants according to the severity of clinical indices

**Variables**	**A** **(Smokers)**	**B** **(Tobacco chewers)**	**C** **(Dual habit)**	**D** **(No habit)**	**Chi-square value**	**P-value**
**Oral hygiene index**					
**Good**	1 (1.33%)	7 (9.33%)	1 (1.33%)	10 (13.33%)	49.96	<0.001*
**Fair**	25 (33.33%)	30 (40.00%)	3 (4.00%)	28 (37.33%)		
**Poor**	49 (65.33%)	38 (50.66%)	71 (94.66%)	37 (49.33%)		
**Gingival index**					
**Mild**	31 (41.33%)	27 (36.00%)	27 (36.00%)	11 (14.66%)	23.97	<0.001*
**Moderate**	43 (57.33%)	41 (54.66%)	46 (61.33%)	53 (70.66%)		
**Severe**	1 (1.33%)	7 (9.33%)	2(2.66%)	11 (14.66%)		
**Probing depth**					
**Shallow**	38 (50.66%)	58 (77.33%)	26 (34.66%)	59 (78.66%)	52.24	<0.001*
**Moderately Deep**	27 (36.00%)	15 (20.00%)	45 (60.00%)	15 (20.00%)		
**Deep**	10 (13.33%)	2 (2.66%)	4 (5.33%)	1 (1.33%)		

*Highly significant (P<0.001).

**Table 4:** Inter-group comparison using post hoc Tukey tests

**Variables**	**A vs. B**	**A vs. C**	**A vs. D**	**B vs. C**	**B vs. D**	**C vs. D**
**OHI**	0.424	0.001*	0.182	0.001*	0.899	0.001*
**GI**	0.002*	0.055	0.001*	0.716	0.519	0.011
**PD**	0.001*	0.620	0.001*	0.002*	0.001*	0.001*
**GR**	0.001*	0.001*	0.001*	0.323	0.001*	0.001*

* Statistically significant (P<0.05). A: exclusive smokers, B: exclusive tobacco chewers, C: individuals with dual habits, D: non-users of tobacco. OHI: oral hygiene index, GI: gingival Index, PD: probing depth (in mm), GR: number of teeth with gingival recession.

### Gingival index

Smokers presented with the lowest mean values of GI among all the groups (Table 2). However, the difference was statistically significant (P>0.05) only for groups B (tobacco chewers) and D (control), respectively. No significant difference was observed between smokers and individuals with dual habits (Table 4). Values were further classified as mild inflammation (up to 1.0), moderate inflammation (1 to 2), and severe inflammation (2 to 3) as presented and compared in Table 3. Despite having poor oral hygiene, all the tobacco users presented with less gingival inflammation than non-users.

### Probing depth

Smokers were found to have the highest average PD among all the groups. All the inter-group differences were statistically significant (P<0.05) except that between group A (smokers) and group C (dual habit) (Table 4). Values were further classified as shallow (<4 mm), moderately deep (4‒6 mm), and deep (>6 mm), as presented in Table 3. Individuals with dual habits were six times, and smokers were 2.25 times more likely to have an average PD of 4‒6 mm than individuals with no habit. Similarly, the odds ratio (OR) for average PD >6 mm was 11.38, 2.03, and 4.17 for groups A, B, and C, respectively (Table 5 and 6).

**Table 5:** Logistic regression analysis for moderately deep periodontal pockets (average PD = 4‒6 mm)

**Groups**	**No. of participants with average PD of 4‒6 mm**	**No. of participants without average PD of 4‒6 mm**	**OR**	**95% CI**	**P-value**
**A**	27	48	2.25	1.07 - 4.69	0.03*
**B**	16	59	1.08	0.49 - 2.39	0.84
**C**	45	30	6.00	2.89 - 12.45	<0.001*
**D**	15	60	1.00		

*Statistically Significant. A: exclusive smokers, B: exclusive tobacco chewers, C: individuals with dual habits, D: non-users of tobacco. OR: odd’s ratio, CI: confidence Interval, PD: probing Depth

**Table 6:** Logistic regression analysis for deep periodontal pockets (average PD>6 mm)

**Groups**	**No. of participants with average PD of >6 mm**	**No. of participants without average PD of >6 mm**	**OR**	**95% CI**	**P-value**
**A**	10	65	11.38	1.41 - 91.35	0.022*
**B**	2	73	2.03	0.17 - 22.84	0.567
**C**	4	71	4.17	0.45 - 38.20	0.206
**D**	1	74	1.00		

*Statistically Significant. A: exclusive smokers, B: exclusive tobacco chewers, C: individuals with dual habits, D: non-users of tobacco. OR: odd’s ratio, CI: confidence interval, PD: probing depth

### Gingival recession

The highest prevalence of teeth with gingival recession was found in group C (12.91%), followed by group B (10.92%), group A (7.52%), and group D (4.8%). All the inter-group differences were statistically significant (P<0.05) except that between group B and group C (Table 4). Compared to the control group, the odd’s ratio (OR) of having gingival recession was the highest (4.33) for individuals with dual habits, followed by tobacco chewers (3.58) and smokers (2.38) as shown in table 7.

**Table 7:** Logistic regression analysis for gingival recession

**Groups**	**No. of teeth with gingival recession**	**No. of teeth without gingival recession**	**OR**	**95% CI**	**P-value**
**A**	153	1878	2.38	1.76 - 3.21	<0.001*
**B**	210	1712	3.58	2.68 - 4.78	<0.001*
**C**	231	1558	4.33	3.24 - 5.76	<0.001*
**D**	63	1839	1.00		

*Statistically Significant. A: exclusive smokers, B: exclusive tobacco chewers, C: individuals with dual habits, D: non-users of tobacco. OR: odd’s ratio, CI: confidence interval, PD: probing depth

## Discussion

The effect of smoking and smokeless tobacco use on the periodontal status can be explained based on two factors: alterations in host tissue and its response to periodontal flora and changes in the microbial challenge. Any of these two might result in a disease pattern different from that in individuals who do not use tobacco. Although smoking has been established as a significant risk factor for periodontitis, the role of smokeless tobacco use is yet to be evaluated adequately in relation to the periodontal disease. In the present study, we tried to bridge this gap in the existing literature regarding the comparison of periodontal status in exclusive smokers with that in exclusive tobacco chewers. However, the evaluation of individuals with dual habits was more important because they have not been studied adequately so far, particularly in young adults. Most previous studies on the periodontal status of smokeless tobacco users have either excluded the group with dual habits^4^ or have not clearly divided the participants into different groups of exclusive current smokers and exclusive current tobacco chewers.^24^

Although tobacco users are found in all age groups, from adolescents to the elderly, it has been reported that in a similar population, most of the tobacco chewers belong to the 21‒41-year age group.^27^ Furthermore, various periodontal conditions are age-related or age-specific. Most of the age-related changes in periodontal tissues are physiological due to decreased immune response and poor remodeling of connective tissues. However, these are also caused by repeated trauma from tooth brushing, abnormal oral habits, iatrogenic damage from repeated scaling and root planing, or due to adverse effects of calcium channel blockers, taken for hypertension, which is very common in individuals after 40 years of age. Therefore, it was appropriate to select a sample that can be expected to be free from these confounding factors so that the effect of tobacco use on periodontium can be evaluated with minimum errors.^20^

Parmar et al reported that quid chewers’ oral hygiene status was significantly worse than non-chewers, irrespective of the oral hygiene measures adopted.^28^ In contrast, the present study showed no significant difference between exclusive smokers, exclusive tobacco chewers, and the control group concerning oral hygiene status. However, participants with dual habits were relatively more careless about their oral hygiene. They showed a significantly higher mean value of OHI than all the other groups, indicating their poor oral hygiene. Katuri et al reported similar OHI values among the three groups of tobacco users.^13^ However, they did not include a control group to compare with. The OHI values of each group were consistent (inversely proportional) to the self-reported frequency of toothbrushing.

Exclusive smokers revealed the least expression of gingival inflammation among all the groups, as reported in most previous studies.^16,17,19^ Despite 65.33% of participants with poor oral hygiene (OHI>3.0), only one (1.33%) among the smokers presented with severe gingivitis. Individuals with dual habits were no different, as 94.66% of them had poor oral hygiene, but only two out of 75 (2.66%) had severe gingival inflammation. Decreased clinical expression of gingivitis in smokers (groups A and C) can be explained based on the vasoconstriction and increased keratinization due to the local effect,^18^ aberrant neutrophil function,^29^ and suppression of pro-inflammatory cytokines.^30^ However, it was interesting to note that, smokeless tobacco users with or without smoking (groups C and B, respectively) also showed lower GI values than the control group. Despite the presence of poor oral hygiene in 94.66% and 50.66% in groups C and B, respectively, <10% of either group presented with severe gingivitis. However, a recent study reported higher GI values in chronic periodontitis patients with SLT use than similar patients without any history of tobacco consumption.^3^ These conflicting data suggest that smokeless tobacco’s effect on the clinical expression of gingivitis needs to be investigated further.

Our study’s data are similar to the findings from most previous studies that indicate a strong correlation between periodontal pockets and smoking.^15,18,31,32^ Surprisingly, a few previous studies have reported similar probing depths in smokers, non-smokers, and those with dual habits.^13,33^ As high as 49.33% of smokers and 65.33% of individuals with dual habits in the present study had an average probing depth of ≥4 mm compared to only 22.67% of exclusive tobacco chewers. Similarly, 13.33% of smokers and 5.33% of those with dual habits showed an average probing depth of >6 mm compared to only 2.67% among tobacco chewers and 1.33% in the control group. More individuals with dual habits showed average probing depth in the range of 4‒6 mm than exclusive smokers or tobacco chewers. Higher average probing depths (>6 mm) were mostly seen in exclusive smokers. The odds of having average probing depth in the range of 4‒6 mm were the highest for individuals with dual habits (unadjusted OR = 6.00) compared to the non-users. The presence of this additive interaction between the two types of exposure (smoking and SLT use) confirms the findings of Mohamed and Janakiram, who also reported that subjects who consumed both forms of tobacco were 3.29 times more likely to have periodontal diseases compared to 1.6 times for smokers and 1.7 times for tobacco chewers.^24^ However, the picture is different when we look at the individuals with an average probing depth of >6 mm. Such cases were significantly associated with exclusive smoking. This finding is similar to one reported by Susin et al.,^32^ who observed that cigarette smokers had a significantly higher occurrence of probing pocket depth ≥5mm than non-smokers, which has also been confirmed by many other studies.^15,31^ However, a recent study with a smaller number of individuals (n=25) and older age (mean age >45 years) reported the average PD to be >7 mm in a group of exclusive SLT users.^3^

It might be speculated that individuals with dual habits exhibit gingival recession and not deep pockets for a similar amount of attachment loss, which is evident in the pattern of gingival recession, noted in the present study. They had the highest number of teeth with gingival recession (unadjusted OR = 4.33), followed by the exclusive tobacco chewers and exclusive smokers. Due to the ambiguity in precisely locating the CEJ, we did not try to measure the extent of gingival recession. Instead, we noted only the presence or absence of it on a given tooth. Similar to our findings, Katuri et al also reported a significantly higher attachment loss among individuals with dual habits and exclusive tobacco chewers. However, the duration of tobacco use was less in both groups compared to the exclusive smokers.^13^ The higher prevalence of gingival recession might be attributed to the physical trauma from the contents of chewable tobacco. A significantly higher prevalence of gingival recession has previously been reported in smokers^34^ and smokeless tobacco users,^19^ but the additive effect of these habits has rarely been observed. The overall poor periodontal status of individuals with dual habits is also evident in the number of remaining teeth, which were significantly lower than any other group. However, the causes of missing teeth could also include non-eruption or extraction for non-periodontal reasons.

The critical strength of the present study was the evaluation of periodontal status by examining all the natural teeth of each participant, unlike most of the previous studies where community periodontal index (CPI)^8,13,35^ or periodontal disease index (PDI)^4^ was used to evaluate only the index (Ramjford) teeth. Since PDI regards periodontitis as an extended version of gingivitis rather than a different disease entity, it is less reliable than a comprehensive periodontal examination.^4^ Further, by including only male subjects, confounding factors such as the effects of puberty and menstruation were avoided. Beedi smoking and SLT use are relatively rare in young adult females in the Indian population.^14,27^ Some previous studies on a similar topic have also focused only on the male population.^19,35^

Although we tried to avoid many confounding factors, some of the observations in our study might remain unexplained. They might vary from other similar studies due to differences in sample size, inclusion and exclusion criteria, parameters, or definition of disease. The lack of socioeconomic data of the participants was another limitation. With a cross-sectional study design, lack of temporal association is an unavoidable drawback. A larger sample size would have yielded more precise results. However, it was not feasible at a single center to make larger exclusive groups with such strict inclusion and exclusion criteria. The radiographic and microbiological evaluation would also yield more information regarding the periodontal disease pattern in these groups.

## Conclusions

Considering the findings of the present study, it is evident that the dual habit of smoking and smokeless tobacco use yields an outcome different from that associated with the exclusive practice of any single habit. Despite having the lowest oral hygiene levels, individuals with dual habits presented minimal gingival inflammation. However, contrary to the exclusive smokers, wherein deep pockets are a common finding, individuals with dual habit presented with relatively shallower periodontal pockets but a significantly higher number of teeth with gingival recession than any other group. Thus, preventing periodontal disease appears to be one more reason for giving high priority to the cessation of smokeless tobacco use in South Asia, which is already burdened with a high prevalence of oral cancer.

## Authors’ Contributions

All the authors have made substantial contributions to the conception and design of the study. AA, AB, and SK were involved in data collection while AA, SK, and MSA performed the data analysis. In addition, all the authors were involved in data interpretation, drafting the manuscript, and revising it critically, and have given final approval of the version to be published.

## Ethics approval

The reported study was approved by the Institutional Ethics Committee.

## Competing interests

The authors declare that they have no competing interests.
